# Are dentists interested in the oral-systemic disease connection? A qualitative study of an online community of 450 practitioners

**DOI:** 10.1186/1472-6831-13-65

**Published:** 2013-11-21

**Authors:** Mei Song, Jean A O’Donnell, Tanja Bekhuis, Heiko Spallek

**Affiliations:** 1 Department of Dental Public Health, School of Dental Medicine, University of Pittsburgh, Pittsburgh, USA; 2 Office of Academic Affairs, School of Dental Medicine, University of Pittsburgh, Pittsburgh, USA; 3 Department of Biomedical Informatics, School of Medicine, University of Pittsburgh, Pittsburgh, USA

**Keywords:** Oral health, Systemic diseases, Evidence-based dentistry, Information needs, Social media

## Abstract

**Background:**

Dentists in the US see an increasing number of patients with systemic conditions. These patients are challenging to care for when the relationship between oral and systemic disease is not well understood. The prevalence of professional isolation exacerbates the problem due to the difficulty in finding expert advice or peer support. This study aims to identify whether dentists discuss the oral-systemic connection and what aspects they discuss; to understand their perceptions of and attitudes toward the connection; and to determine what information they need to treat patients with systemic conditions.

**Methods:**

We retrieved 14,576 messages posted to the Internet Dental Forum from April 2008 to May 2009. Using natural language processing and human classification, we identified substantive phrases and keywords and used them to retrieve 141messages on the oral-systemic connection. We then conducted coding and thematic analysis to identify recurring themes on the topic.

**Results:**

Dentists discuss a variety of topics on oral diseases and systemic health, with the association between periodontal and systemic diseases, the effect of dental materials or procedures on general health, and the impact of oral-systemic connection on practice behaviors as the leading topics. They also disseminate and share research findings on oral and systemic health with colleagues online. However, dentists are very cautious about the nature of the oral-systemic linkage that may not be causal. Nonetheless, they embrace the positive association as a motivating point for patients in practice. When treating patients with systemic conditions, dentists enquire about the cause of less common dental diseases potentially in relation to medical conditions in one-third of the cases and in half of the cases seek clinical guidelines and evidence-based interventions on treating dental diseases with established association with systemic conditions.

**Conclusions:**

Dentists’ unmet information needs call for more research into the association between less studied dental conditions and systemic diseases, and more actionable clinical guidelines for well-researched disease connections. To improve dissemination and foster behavioral change, it is imperative to understand what information clinicians need and in which situations. Leveraging peer influence via social media could be a useful strategy to achieve the goal.

## Background

Oral health is essential to a person’s overall health and well-being [1]. However, practicing dentists in the United States see an increasing number of patients in ill health with comorbidities that complicate care. Comorbidities occur, in part, because patients are aging and disease epidemics, such as obesity and diabetes, are worsening. Such patients are challenging to care for when the relationship between oral and systemic disease is not well understood. Moreover, the high prevalence of professional isolation of dentists in the US [2] exacerbates the problem. Because most dentists work as solo practitioners, they rely on colleagues (when possible), textbooks, and personal journal collections as their preferred sources of clinical information [3,4]. However, the geographic distance and lack of time during clinical care often make communication with other dental colleagues poorly coordinated, inconvenient, and ineffective. Similarly, timely communication with patients’ physicians about related medical conditions can be difficult. Professional isolation also limits quality assessment of care. Since most dentists practice in isolation, few standardized measures of meaningful treatment outcomes have been developed for and adopted across practices. As a result, comparison of patient outcomes across dental providers or delivery systems is very challenging [5].

In recent years, the relationship between oral and systemic disease (henceforth referred to as the oral-systemic connection) has become a leading research topic. Researchers have looked at the potential mechanisms by which oral bacteria may contribute to systemic inflammation [6,7]. Numerous epidemiological studies investigated the association between oral diseases and myriad systemic conditions, including cardiovascular diseases [8-10], diabetes [11-13], pneumonia [14], rheumatoid arthritis [15,16], and pregnancy outcomes [17,18]. Intervention studies focused on the relationship between dental treatment and its impact on certain systemic conditions, such as the effect of periodontal treatment on the incidence of cardiovascular events [19] or glycemic control [20,21].

While many studies suggest a positive and independent association between oral and systemic conditions [8,10-16], particularly between periodontal disease and heart disease and diabetes mellitus, the nature of this relationship remains controversial. A critical review by the American Heart Association (AHA) concluded that periodontal disease is independently associated with atherosclerotic vascular disease (AVD); however, current evidence does not support any causal relationship [22]. Similarly, the benefits of periodontal therapy on local periodontal inflammation have been supported, but not on long-term systemic inflammation. Multiple clinical studies suggested the association between periodontal disease and diabetes is bidirectional, and two systematic reviews concluded that periodontal therapy produced a modest improvement of glycemic control [23,24].

Despite the growing literature on the oral-systemic connection, little is known about the knowledge and attitudes of practicing dentists about the connection, their information needs on this topic and if research findings guide their practice behaviors. Only a few surveys looked at dentists’ knowledge of the connection, but primarily about diabetes. In one survey, while the majority of general dentists obtain information from new patients about their history of diabetes, just 60% discuss oral-diabetic issues with patients, and a mere 15% monitor patients’ blood glucose levels or regularly communicate with patients’ physicians [25]. Similarly, another survey of Delta Dental (a dental insurance provider) dentists found that 86% advise diabetic patients about periodontal risks, but only 18% provide diabetic-related services [26]. As for informing practice, only one clinical guideline by the AHA on the prevention of infective endocarditis [27] appears to be well established and generally adopted by dentists.

While these studies tried to quantify dentists’ knowledge of the connection, their focus is narrow. Additionally, the research is limited methodologically by its reliance on self-reported answers to forced-choice survey questions, which falls short of revealing in depth what dentists actually know or want to know about this topic. The objectives of this qualitative study are to identify whether practicing dentists discuss the oral-systemic connection in a manner that eliminates observation bias, and if so, what aspects they discuss; to understand their perceptions of and attitudes toward the connection; and to determine what information they need to treat patients with systemic conditions. Given the prevailing professional isolation in dentistry, we believe that understanding what practicing dentists discuss, what questions they raise, as well as whom they ask for advice, can help us develop informatics applications for timely delivery of useful research findings at the point of care. In this way, we may change practice behavior and ultimately improve patient outcomes.

## Methods

### Materials

We retrieved messages posted to the Internet Dental Forum (IDF), one of the oldest online forums for dentistry launched in 1994. IDF includes approximately 450 subscribers worldwide, mostly dentists from North America directly involved with patient care. Dentists join this forum to share knowledge and experience, to ask for advice on diagnosis and treatment for specific patient cases, and to discuss other issues in dentistry and related topics. Studying IDF messages posted from April 2008 to May 2009 allowed us to explore what dentists discuss and practice in an unobtrusive and natural way. This study was approved by the University of Pittsburgh Institute of Research Board (PRO08040313) with the permission from IDF to extract and analyze the message contents for research and publication.

To find a manageable subset of messages for this study in a large corpus of 14,576 messages, we first developed a method based on natural language processing (NLP) and human classification [28,29]. After de-identification and extensive preprocessing, we used the Natural Language Toolkit [30] to identify substantive phrases and keywords based on point wise mutual information scores. Two clinical dental researchers (HS and JAO) classified collocated phrases into 13 broad categories with subcategories using an iterative, consensus-based process (see Additional file 1). This categorization mapped the topical domain for the dentists who posted to the IDF during the study period [28]. We saved categorized messages in a reusable database for future content analyses.

We developed a plan for targeted retrieval of relevant texts from our database of processed messages. We used substantive phrases and keywords identified in the earlier study as search strings to retrieve messages about the oral-systemic connection [28]. We purposefully selected all the messages in three of 13 categories in which dentists were mostly likely to have discussed oral and systemic disease. The categories were Systemic Disease (n = 299 messages), Periodontics (n = 54 messages) and Research (n = 100 messages). After deduplication, we retrieved 437 messages for content analysis in this study.

### Coding procedures

The two clinical dentists (HS and JAO) and one qualitative researcher (MS) coded the messages. As a first step, we randomly sampled 20 messages (4.6%, 20/437) from our database using NVivo v.9 (QSR International, Australia), a software application for analyzing qualitative data. Using this development subset, we created general codes based on our research questions, such as *oral-systemic connection* and *information needs*, to guide subsequent coding. Following coding guidelines developed by the team, the qualitative researcher categorized all messages and coded in depth those on oral-systemic connection with open coding, the process of selecting and naming categories and identifying commonalities of the data. Afterwards, we randomly selected 25% of all coded messages for independent review and validation by the two dentists. The team then discussed coding results and resolved any discrepancies between the clinicians and the qualitative researcher.

### Data analysis

Data analysis was conducted using NVivo v.9. We used the thematic analysis method [31] to identify common themes or patterns in the data regarding various aspects of the connection between oral and systemic diseases. In each message, excerpts were first selected and assigned a relevant code or category (e.g., information need). Then all message excerpts with the same code across messages were merged and collated into one category. Within each code (category), the qualitative researcher reviewed each excerpt by constant comparison method to identify commonalities and differences, and then summarized the commonalities to come up potential themes. The whole team reviewed and discussed these themes to ensure that they were valid in relation to both the coded message excerpts and the entire sample of messages.

## Results

**Figure 1:**
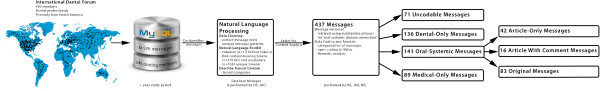
Flowchart of the preprocessing, retrieval and selection of messages in this study.

We retrieved 437 messages posted by 90 IDF participants (85 from US and 5 worldwide based on the posting email addresses used), among whom 9 people posted 220 messages, making up 50% of the messages. Figure 1 describes how the messages were processed, retrieved and selected for final coding. After an initial reading, we categorized the messages into four groups: oral-systemic connections (141 messages), dental topics other than oral-systemic connection (136 messages), medical topics other than oral-systemic connection (89 messages) and uncodable messages that were outside the scope of dentistry and medicine or were too fragmented to code (71 messages). Given the focus of our study, we further coded in depth the 141 messages on oral-systemic connections. The major themes are described below.

### Discussion on oral-systemic connection

Dentists posted messages on a variety of topics involving the relationship between various oral and medical conditions or medications. Table 1 summarizes general topics and the number of messages coded for each topic. In order of frequency, the three most discussed oral-systemic topics were association between periodontal diseases and systemic health, dental materials or procedures and general health, and general discussion on the oral-systemic connection. In comparison, less discussed topics included medical emergencies in dental offices, management of medical problems through dental treatment and premedication for medical conditions during dental treatment. A description of major themes that emerged from these topics is presented below.

**Table 1:** Summary of oral-systemic connection topics organized by number of messages and excerpts coded

**Topics on oral-systemic connection**	**Messages with this code n (%)**	**Excerpts with this code n (%)**
Periodontal diseases and systemic health	48 (34)	61 (34.3)
Dental materials or procedures and health	25 (17.7)	28 (15.7)
Other oral conditions and systemic diseases	21 (14.9)	30 (16.9)
General discussion on oral-systemic connection	21 (14.9)	26 (14.6)
Management of medical emergency in dental offices	11 (7.8)	14 (7.9)
Management of medical problems through dental treatment	8 (5.7)	12 (6.7)
Premedication for dental treatment due to systemic conditions	7 (5.0)	7 (3.9)
Total	141 (100)	178 (100)

#### Connection between oral diseases and systemic conditions

Among all messages on oral diseases and systemic health, discussion on periodontal and systemic diseases ranked number one with 48 messages, of which a predominant majority revolved around the connection between periodontal and heart diseases (30 messages), followed by periodontal conditions and pregnancy outcomes (4 messages) and diabetes (3 messages). Dentists focused their discussion of periodontal and heart diseases on potential linkage mechanisms, reflecting on some research findings. For example, one dentist embraced the theory of a common inflammatory defect:

There is good evidence that refractory perio disease is a genetic disease, a defect in the inflammatory system … This same inflammatory defect leads to coronary artery disease and premature deliveries. All cause an increase in the C reactive protein. Each is a marker for the other but you cannot cure one by treating the other. Decreasing the CRP does not mean you cleared the other.

Another dentist shared his view on the connection with a different interpretation:

Suspect a bigger source of major issues comes from bacterial toxins stimulating the immune system which then activates the matrix metalloproteinases. These collagenase enzymes attack the intra-arterial plaques, since they are loaded with these same bacteria and their marker toxins thanks to years of chronic gum leakage. This, then, cleaves the base of the atherosclerotic plaque which then fully comes loose, makes a thrombus or acts like a flapper valve thus closing off arterial flow.

While a majority of the posts agreed on the existence of an association between periodontal and heart diseases, two dentists argued for no association from their personal or clinical experience. One said:

The man had a myocardial infarction that totally blocked a major coronary artery and killed him. The same thing is likely to happen with me and I get regular dental care and cleanings every three months. It ain’t got crap to do with perio. I had a 98% blockage on the left descending coronary artery and was walking around dead. There was little doubt that mine was due to damned 20 years of smoking.

In addition to periodontal diseases, dentists talked about other oral diseases correlated with medical problems, such as the impact of missing teeth, root canal treatment and sleep apnea on general health. Dentists also paid increasingly more attention to other factors that played a role in causing certain oral conditions, such as genetics, chemotherapy and medications. One dentist described his observation of some orthodontic conditions in Africa:

I got into isolated villages where there was an obvious genetic component to some orthodontic situations. You would see child after child with malformed Cl 3 [where the lower jaw protrudes ahead of the upper jaw]) skeletal problems and less frequently Cl 2 skeletal [where the lower jaw is retruded or too short relative to the upper jaw]. I have noticed that certain African tribes also have widespread skeletal maloccusions.

#### Impact of dental materials or procedures on general health

Dental materials or procedures and general health was another favored topic of the dentists’ posts, including two controversial topics: anesthesia complications of third molar extraction and toxicity of mercury in dental amalgams. Most dentists maintained that the removal of third molars is safe in most situations, even with occasional media reports of death from the procedure. However, they did adopt a more cautious attitude towards removing them unnecessarily, as one dentist commented:

If they can maintain a second molar the thirds can also be maintained. If they cannot, if they get recurrent pericoronitis, get them out. If the pericoronitis is resolved with warm rinses and cleaning, let them erupt. It is not pathologic to have 3rd molars. Do not take them because they might be a problem. The best science says 10% maybe 15%, not 100% need to be removed.

The discussion on mercury was stimulated by a statement on the FDA website in 2008 (http://www.nbcnews.com/id/25139198/#.USPHjvJkrh4) suggesting that mercury contained in dental amalgams may pose neurological risk to children and pregnant women. Dentists generally thought the warning was taken out of context. Mindful of the potential risk of amalgams, most of them remained committed to using it in certain situations. One example was offered by this dentist:

There is certainly a downside in the amalgam scare. Although I use amalgam sparingly now, I would hate to give it up completely for certain situations. I don’t think that half the dentists can place a satisfactory composite resin without sensitivity and with good contour and contacts.

#### Impact of oral-systemic connection on dental practice

For broader discussions of the oral-systemic connection, the leading topic was its impact on dentistry generally and on practice specifically. In the context of an aging population with more comorbidities, dentists repeatedly brought up the concepts of “age relevant dentistry” or “holistic dentistry” which emphasizes taking care of the patient’s whole body rather than treating isolated organs or systems, as one dentist proposed:

We need to take a different approach to their care from the traditional drill, fill and bill. The snatch them out, sew them up, and let them try to get used to “china clippers” may not be the best approach. We need to balance life expectancy, modified by medical and physical condition, cognitive ability, against traditional dentistry, cost, lack of any third party coverage.

Another dentist echoed the previous comment, adding the expectation that a holistic dentist may practice as a primary care provider in the future:

Mounting research supports what holistic dentists have long believed: Although the mouth may not be the window to your soul, it can reveal potential medical problems in other parts of the body, including the heart, lungs and brain. And as the connection grows stronger, dentists who focus on treating the whole body as well as the teeth and gums with prevention, education and nutrition playing a central role may one day assume the role of a “primary care doctor”.

In terms of changes in daily practice, some dentists have been applying a more holistic approach to take into account various factors when treating patients, such as medications, nutrition and health behaviors. They mentioned that other “alternative” dentists might use biocompatible dental materials, natural products such as herbal toothpastes and mouth rinses, as well as herbal or homeopathic remedies for pain, irritation and infections.

While promoting changes in dental practice, some dentists strongly believed that educating patients about the oral-systemic connection is a crucial component in delivering optimal care, even when strong evidence in research does not yet exist. Coupled with false claims, irresponsible advertisement and misinformation, patient education remained a real challenge. For example, a dentist voiced dissatisfaction with some advertising to patients that implied that periodontal diseases can cause heart disease:

From a couple of questions by patients in the office that I’ve answered, I know that people are confused, and in the light that some cardiac patients whom are looking for the “easy” way out, this could be how their brains interpret the information.

#### Management of patients with medical comorbidities

Another thread of discussion was centered on how dentists should apply their knowledge of the oral-systemic connection to provide dental care to patients with medical comorbidities, predominantly cancer patients undergoing radiation or chemotherapy. These preventive or treatment-oriented services could help prevent certain dental conditions after the treatment, such as infection from exposed roots, dental abscesses and large carious lesions, or manage ongoing problems, such as radiation-induced xerostomia (dry mouth syndrome). This approach has led to very positive outcomes for a number of cases presented on the forum, as illustrated by a dentist providing preventive care to a cancer patient during chemotherapy:

…I am currently seeing things in the preventative dental world that I live in. I have a cancer patient that is 5.5 years out and still has his teeth. He was told by his MD he would lose them within 18–24 months. Both the patient and I think it is the early preventative Rx-ing of MI Paste [a calcium, phosphate, and fluoride tooth treatment to protect teeth from decay] during his chemo and every day since that has kept his teeth so healthy.

Sometimes, even if there was not a clear connection between a dental and medical condition, a dentist’s clinical experience and intuition lead to successful management of medical problems. One such example was offered by a dentist when treating a young patient with arthritis and fibromyalgia:

I had a really great result this week. A 16 year old who has arthritis, and she apparently also has fibromyalgia symptoms. Headaches all night, they also came on during the day. No promises etc., but I felt that it would be a good go. Made her a night time NTI [a removable mouth splint used for headache relief] and a lower “Bookmark” for day time. She came in yesterday for a 2-week follow up. All smiles, no headaches, worked almost instantly, loves the day time one as it is comfortable, works and doesn’t show :) makes my day for sure :)

Aside from concerns regarding good management of patients with comorbidities, another was how to deal with medical emergencies in their practices. For example, people with adrenal insufficiency may experience an adrenal crisis under stress during dental treatment. A dentist offered his solution:

A number of patients with limited adrenal insufficiency may appear healthy but will experience acute episodes when under stress. Shock and fever may be the only signs. Give hydrocortisone in this case. Sodium and water requirements will be less in this case but managing fluids on sick patients is not something I’d do....

For more routine preparation for medical emergencies, dentists shared what they do in their practices, such as what drugs and instruments should be included in an emergency kit; if and when to call 911; and whether a defibrillator should be installed in the office. They seemed to agree on preparing and refreshing an emergency kit regularly, but less on buying a costly defibrillator for every dental office.

### Attitude towards the oral-systemic connection

In general, most posting dentists believed that there is a real connection between oral and systemic diseases, particularly the association between oral inflammation and the inflammation of the whole body, as one dentist said:

There may not be definitive research, but it stands to reason that inflammation and infection in any part of the body cannot be healthy for the rest of the body. It has got to compromise general immunity.

While cognizant about the connection, many dentists also realized that oral inflammation is one of multiple factors that may play a role in causing a specific disease, as one dentist talked about the risk factors for cardiac disease:

I guess I look at it this way- cigarettes have a role, diabetes has a role, and high blood pressure has a role and we take these things very seriously. If the top doctor in the country says that gum disease clearly has a role in cardiac disease, I think it is time to take it as seriously as cigarettes, diabetes, high blood pressure, and a heart attack.

Informed about the oral-systemic linkage, many dentists on the forum were very cautious about the direction of this relationship and remained suspicious towards any discussion of a causal relationship. They were still waiting for strong scientific evidence. One dentist said:

As professionals we have an obligation to use the best science that is available when we advise patients. There is good science to back my stance and none to show that treating perio will prevent heart disease, or death from cardiovascular disease.

A few dentists placed extra importance on the issue of causality and discussed its ethical ramifications. For example, some believed that it is unethical to imply the relationship is causal. One dentist stated his opinion this way:

What we have here is [a] problem with ethics. Periodontal disease may predispose patients to cardiac, st[r]oke and diabetic troubles. There is no solid science to support causation. The problem is the unethical presentation of perio as a causal condition. It is repugnant and a slur on our profession to proselytize an unsupported suspicion.

For those trying to proselytize the causal link to patients, dentists warned that the practice has the danger of misleading patients, as one dentist commented:

Can you guarantee a patient that treatment of periodontal disease will have an impact on heart health? I find it very “wishy- washy” to tell patients that “there is a relationship between” the two disease processes. I still mention it to patients but honestly, it comes across like a scare tactic if you place too much emphasis on it.

However, in absence of a definitive answer from the scientific community, dentists on the forum chose to embrace the association and tried to use this as a motivating point for patients to pay more attention to their oral health.

I agree that there is no sound science to the perio/cardiac cause-and-effect. But what is the downside of treating and preventing perio disease? I try to use the possibility to leverage patients into better home care and frequent recalls.

### Dissemination of knowledge on the oral-systemic connection

In addition to informing themselves about the oral-systemic connection, dentists also shared scientific studies from academic journals and articles from mainstream media to disseminate knowledge to their peers. Dentists posted 58 messages that contained research studies, abstracts, or magazine articles, with or without further comments. The posted articles covered a variety of topics on the oral-systemic connection. Taken as a whole, these messages comprised 41% of the 141 messages included in the analysis.

Table 2 categorizes 58 messages with posted articles according to article topic. The title of a sample article for each category is also provided. The articles addressed a variety of oral-systemic connections, with the association between periodontal diseases and systemic diseases as the leading topic (55%, 32 messages), followed by dental materials and systemic health (7%, 4 messages), general discussion on the oral-systemic connection, complications of dental anesthesia procedures and dental medication on systemic health (5% each, 3 messages). In general, the results were consistent with the leading topics discussed by dentists in their own postings described earlier.

**Table 2:** Classification of messages posting articles on the oral-systemic connection organized by article topic (n = 58 messages)

**Topic**	**Title of sample study**	**# articles (%)**
Periodontal diseases and systemic health	*Relationship between periodontal infections and systemic diseases*[32]	32 (56%)
Dental materials or procedures and systemic health	*Gooping on denture cream can be health hazard*[33]	7 (12%)
Other oral conditions and systemic diseases (e.g. missing teeth, sleep apnea, etc.)	*Heavy snoring as a cause of carotid artery atherosclerosis*[34]	7 (12%)
General discussion on oral and systemic health	*Seniors’ teeth a growing concern: Many lack coverage, money, specialists*[35]	3 (5%)
Oral medication and systemic health	*Fluoride and its effect on human intelligence. A systematic review*[36]	3 (5%)
Oral condition as a result of medical treatment	*Oral complications of chemotherapy and head/neck radiation*[37]	2 (3%)
Management of medical emergency in dental offices	*Why put a defibrillator in a dental office? *[38]	2 (3%)
Oral abscess caused by dental plaque	*Experimental Abscess Formation Caused by Human Dental Plaque*[39]	1 (2%)
Virus activity and oral diseases	*Herpes viruses in endodontic pathoses: Association of Epstein-Barr Virus with irreversible pulpitis and apical periodontitis*[40]	1 (2%)

Regarding the sources of posted articles, 38 (65%) out of 58 were published in peer-reviewed journals (e.g., *Sleep; Journal of Endodontics*), 11 (19%) were from an institute or an organization’s website (e.g., National Cancer Institute; Food and Drug Administration), and 9 (16%) were published in print or online newspapers (e.g., Reuters; Baltimore Sun).

### Information needs on treating patients with systemic diseases

As previous sections show, dentists posted thoughts and opinions on a spectrum of issues regarding the oral-systemic connection. They not only talked about these topics, but also posted questions or made inquiries to colleagues regarding a specific dental question in connection to systemic conditions. These information needs were driven by various situations or factors, such as when they faced a dental patient with medical conditions; when they read an article in an academic journal or mass media outlet; or when they encountered a question during a discussion with a colleague or at a continuing education event.

As for what information they sought, the need varied depending on the individual case. Among 37 situations in which a dentist asked an oral-systemic connection question, 13 (35%) were inquiries on the cause or diagnosis of a disease, 10 (27%) on evidence and guidelines, and 8 (22%) on treatments and interventions. The remaining 6 (16%) asked how to meet patients’ needs or regarded education and training in dental offices.

#### Looking for cause or diagnosis of diseases

While there is abundant research on the relationship between certain oral and systemic conditions, such as periodontal disease and diabetes or heart disease, dentists seemed to want more information about the relationship between less common oral conditions and other medical problems or medications. Most of the information needed by dentists on the cause of diseases fell into this category. For instance, one dentist asked about the potential relationship between a painful portion of exposed bone on the tongue side of the lower left 3rd molar (wisdom tooth) and the medication methotrexate [a drug used to treat certain cancers, severe psoriasis, or rheumatoid arthritis] for a female patient:

I referred her to an oral surgeon, who reduced the exposed bone and considers the area will granulate over and heal. Is it possible the methotrexate was a causative factor, or just coincidence?

Another dentist pondered about a possible diagnosis of recurrent ulcers on a patient’s palate by posting the following case:

The ulcers follow the distribution of the greater palatine nerve, so I suspect herpes zoster. Most of the time I've seen this it's been unilateral, but in his case it’s always bilateral. What other diagnoses should I be considering, or does herpes zoster sound the most plausible because of the distribution along the nerve trunk?

### Looking for evidence and guidelines

In some cases, when there was previous knowledge on the link between a dental and medical condition, such as mercury and neurological risks or porphyria and anesthesia, dentists inquired about new research while others looked for clinical guidelines on how to treat a patient. One dentist brought up a case of his new patient with Marfan syndrome who was premedicated by her previous dentist. Unable to find a reference, this dentist turned to colleagues on the forum:

It is late right now, and I can’t find a reference that says “premedicate”. Don’t think it is needed. I know, flame away, but anyone knows?

#### Looking for treatment and interventions

In a third group of cases, dentists were caring for dental patients with comorbidities. These dentists needed to develop a treatment plan while taking into account patients’ medical problems. With limited experience, they posted their plans to seek feedback and reassurance from their online colleagues. In one case, a dentist was treating a patient with moderate Alzheimer’s who needed to have two teeth replaced on the upper right side of the mouth. Given that the patient was somewhat cooperative and non-combative, the dentist considered using a valplast appliance [a flexible, non-metal removable partial denture]. Having never placed such an appliance himself, the dentist actively sought his colleagues’ suggestions. For another patient who came for an oral examination before radiation treatment but after oral cancer surgery, the dentist posted his exam findings and consultation with the radiologist to seek comments:

My exam found heavy calculus with pockets to 7 mm and recurrent decay in #2mo [the chewing and interproximal surfaces of the upper right 2nd molar]. This may result in root canal treatment. I called the radiologist and told him of my findings and asked him if he would reach the upper right with the radiation. He said only minimally. I then said I would thoroughly debride the calculus, do the restoration and have one follow up visit to check healing before the radiation. Any comments?

## Discussion

Dentists on the IDF discussed a wide range of topics related to the oral-systemic connection, from general discussion of the connection from a public health perspective to dental amalgams and neurological risk. Moreover, many were cognizant of current research. The association between periodontal diseases and various systemic conditions and its nature was the most discussed topic, consistent with the leading topic in the research community. Additionally, dentists disseminated research findings to their online colleagues, with the majority of the articles still on periodontal diseases and systemic health. Most of the shared articles were published by peer-reviewed journals or by reputable websites and mainstream media. Thus, the shared information should be considered valid. All of these points suggest that in a relatively isolated practice environment, an online community could serve as a useful venue for dentists to learn, discuss and share new scientific discoveries that may guide or inform their daily practice behaviors.

In light of the oral-systemic connection, IDF dentists were open to new practice approaches. They advocated the concept of “age relevant dentistry” and a more holistic approach to taking into account multiple risk factors and medical conditions when treating dental patients. Some also suggested that in a new practice environment dentists could and should play a bigger role in delivering optimal patient care, a position also proposed by some researchers [41,42]. One such scenario for dentists to actively practice team-based care with physicians could be in the screening, monitoring and co-management of diabetic patients in dental offices.

While embracing the positive impact of knowing about oral-systemic connections on dentistry, IDF dentists were very cautious about making false claims about the nature of presumed relationships and warned against misleading patients and making inappropriate treatment decisions, especially for patients suffering from debilitating diseases such as heart disease. This indicated that dentists are adopting an ethical approach regarding diseases and major systemic conditions when the literature is replete with studies suggesting positive associations. Nevertheless, even when research is not definitive, dentists still want to leverage awareness of possible connections to motivate patients to practice good oral health. This intention reflects their pragmatic practice approach to dentistry. For better clinical outcomes, patient education was considered a key component, but was perceived as missing or inadequate in current practice. This finding suggests that we need to design and implement effective patient education programs on the oral-systemic connection for delivery in practice environments.

Dentists on the forum also had some unmet information needs in daily practice when treating dental patients with systemic conditions. In one third of the cases, they searched for the cause of a less common dental disease, potentially in relation to a medical condition. This attests to the need for researchers to explore further the connection between non-periodontal dental disease and other medical conditions, and to better inform dentists about their findings. In half of the cases, dentists looked for clinical guidelines or appropriate interventions to treat a dental patient with medical comorbidities. This information need speaks to the urgency for the clinical community to develop and disseminate guidelines on well-researched oral-systemic connections to guide clinical care. Surprisingly, dentists had few information needs for patients with heart disease, diabetes or periodontal diseases, conditions at the core of the oral-systemic connection. One explanation could be that dentists who keep up with the research literature might already follow clinical recommendations proposed by some articles and commentaries [43,44].

When established guidelines do not exist on treating dental patients with medical conditions, especially for those with less studied diseases, dentists in this study took actions based on their clinical experience in some cases, but sought colleagues’ advice online in others. This is consistent with other studies [45] and highlights the importance of peer influence. In certain situations, dentists seem to value peer recommendations more than recommendations in published research or evidence-based guidelines.

As for what motivated dentists to ask questions on the oral-systemic connection, dentists initiated questions when they came across a challenging patient, heard about a patient case from a colleague or read a relevant research article. However, it is unclear in the messages regarding a patient case if the patient raised the question initially before the dentist posted it on the forum. Additional research with dental patients is needed to assess their knowledge and information needs of oral-systemic connection in order to inform patient education on oral and general health.

As far as we know, this is the first study to examine knowledge, attitudes and information needs of practicing dentists regarding the oral-systemic connection by conducting an in-depth analysis of their online messages. Based on our findings, researchers could explore how peer influence might be used to improve dissemination of evidence-based medicine and dentistry to colleagues, as well as change behavior in daily practice. For example, information about effective interventions and best practices could be delivered by peers to educate peers via social media and virtual social networks. In this way, future developers could reduce the well-known gap between research discovery and program delivery in healthcare settings where the provider is likely to work in relative isolation.

This study has a few limitations. A major one is that we used a single source to mine electronic messages. As a result, messages from a convenience sample of dentists worldwide might not represent dentists practicing in the U.S. Further, clinical topics of interest to one dental online community might not fully reflect those to other dentists in the U.S. Secondly, participation inequality existed on the discussion forum [45]. In this study, a small group of active participating dentists posted half of all the messages analyzed. However, this was not a concern since we were more interested in identifying the scope and type of knowledge on the oral-systemic connection discussed and disseminated on the forum and less on who posted the messages. The skewed distribution of participation is also typical of other virtual communities [45]. A third potential limitation was that the messages were retrieved from a selected period and topics might change over time. However, we purposely selected a study period when the oral-systemic connection was a leading research question, so the time period was especially useful in exploring this topic. Nevertheless, it will be helpful in the future to sample another period to compare and contrast the results.

## Conclusions

Dentists who are members of this online community discuss a variety of topics about the association between oral and systemic diseases. Many are cognizant of major research findings and some use the forum to disseminate and share findings with colleagues. While open to changing their practice behaviors based on presumed associations between oral and systemic diseases, dentists are very cautious about the nature of relationships that may not be causal. Their unmet information needs call for more research into the connection between less studied dental conditions and systemic diseases, and more actionable clinical guidelines for well-researched disease connections. Previous attempts to disseminate research findings by simply providing information rarely changed clinical practice [46]. To improve dissemination and foster behavioral change, it is necessary to understand what information clinicians need and in which situations. Moreover, an awareness of how they share knowledge with peers will inform the development of more efficient and effective interventions to translate scientific discoveries into daily practice. To this aim, leveraging peer influence in a social network via social media could be a useful strategy.

## Abbreviations

ADA: American Dental Association; AHA: American Heart Association; AVD: Atherosclerotic vascular disease; IDF: Internet Dental Forum; NLP: Natural language processing.

## Competing interests

The authors declare that they have no competing interests.

## Authors’ contributions

All authors were involved in the conception and design of the study. TB preprocessed the messages and identified topics in the larger corpus. MS, HS and JOA performed analyses and interpreted the data. All authors participated in either drafting or critically revising the manuscript and approved the final manuscript.

## Pre-publication history

The pre-publication history for this paper can be accessed here:

http://www.biomedcentral.com/1472-6831/13/65/prepub

## Supplementary Material

Additional file 1 **Classification of dental phrases with keywords in blue small caps.** Table of keywords by category displayed at the end of the classification. Click here for file
